# Placing Locking Compression Plates as an External Fixator in Wild Animal (Crocodile) Bite Victim: A Case Report

**DOI:** 10.7759/cureus.47511

**Published:** 2023-10-23

**Authors:** Fathima S Mubarak, Kandeepan Kanagaratnam

**Affiliations:** 1 Cardiothoracic Surgery, Hull University Teaching Hospital, Hull, GBR; 2 Surgery, Ministry of Health Sri Lanka, Colombo, LKA; 3 Orthopedic Surgery, Ashraff Memorial Hospital, Kalmunai, LKA

**Keywords:** open tibia fracture, dynamic external fixation, locking compression plates, distal tibia fracture, crocodile jaw

## Abstract

Animal bites can cause severely contaminated open fractures, especially in the hand, wrist, and lower extremities, requiring expert orthopedic care. This case report emphasizes the unique issues posed by such injuries, particularly in low-income areas with limited access to comprehensive medical services. Pediatric examples, such as open tibiofibular fractures caused by animal encounters, highlight the significance of individualized treatment techniques. Crocodile bites, though infrequent, present an extreme peril and potential fatality. In Sri Lanka, where various crocodile species inhabit the waters, such incidents are rare but present. Long bone fractures caused by crocodile bites are significant and complicated injuries. The enormous force of a crocodile's jaw can cause substantial damage to bones, tendons, and surrounding tissues, often resulting in significant bone and soft tissue loss. Managing such injuries is extremely difficult, especially in low-resource settings. The use of a locking plate as an external fixator is a novel approach in the treatment of open fractures, nonunion, septic arthritis, and even as a distraction osteogenesis adjuvant. While it is not as common in typical fracture fixation textbooks, it is critical in specialized instances. This study describes a unique case of a 13-year-old boy with a Gustilo-Anderson Type IIIA crocodile bite who was treated in a low-income environment with a locking plate as an external fixator.

## Introduction

Animal bites can lead to severely contaminated open fractures, posing unique challenges in orthopedic intervention. These injuries, often involving the hand, wrist, and lower extremities, require specialized care. In low-income settings, limited access to comprehensive medical facilities may exacerbate complications. Pediatric cases, such as open tibiofibular fractures following animal encounters, emphasize the need for tailored treatment strategies. Additionally, unusual cases, like hyena bite injuries, underscore the diverse nature of such incidents. Specialized interventions, such as in cases of dog bite wounds to the hand, are crucial for optimal recovery. Understanding these specific fracture patterns resulting from animal bites is essential in guiding treatment approaches and ensuring favorable outcomes for affected individuals [1].

Crocodile bites, while relatively rare, can be extremely dangerous and potentially fatal. In Sri Lanka, where various species of crocodiles inhabit the waters, such incidents are infrequent. A five-year survey from 2008-2012 recorded only 33 cases of crocodile attacks on humans, with the majority being attributed to saltwater crocodiles. These attacks resulted in eight fatalities. It's worth noting that crocodile attacks tend to occur during activities such as bathing, washing clothes, or swimming [2].

Long bone fractures resulting from crocodile bites are serious and complex injuries. The immense force exerted by a crocodile's jaw can cause extensive damage to bones, tendons, and surrounding tissues. These are severely contaminated and often with significant bone and soft tissue loss. Managing such injuries is a real challenge, especially in a low-resource setting.

External plate attachment is not a unique concept. Although it has been used to treat open fractures, nonunion, septic arthritis, and even as a distraction osteogenesis adjuvant, it is still considered unique and does not have the same place in traditional fracture fixation textbooks as other treatments [3].

Here we present a unique case of crocodile bite in a 13-year-old presenting with Gustilo-Anderson Type IIIA which was managed and treated with a locking plate as an external fixator in a low-income setting [4].

## Case presentation

**Figure 1:**
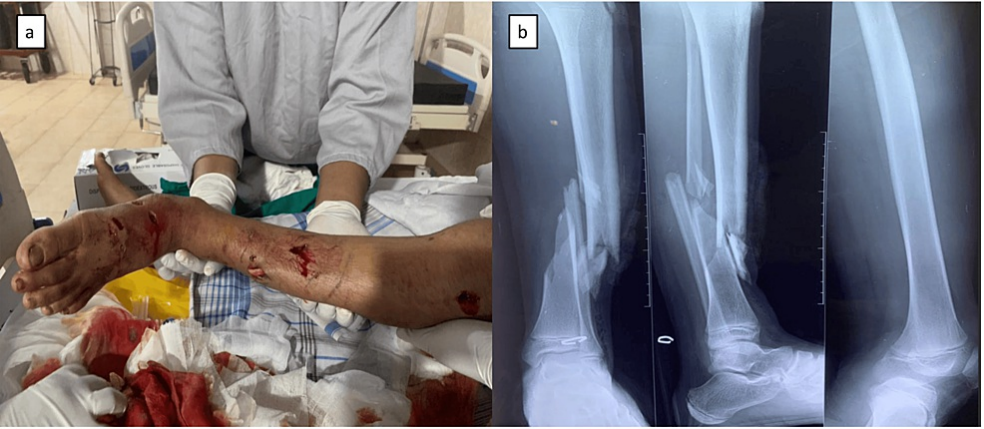
Immediate presentation to the hospital with obvious deformity of the lower limb and bite marks a) Skin breaches and bite marks of the crocodile with bone protruding from the skin; (b) Gustilo-Anderson Type IIIA left distal tibial fibula open fracture

A 13-year-old boy was taken to a base hospital in rural Sri Lanka after being bitten by a crocodile on the lower extremities. Although crocodile bites are infrequent in this area, they do occur. This patient was referred from the general surgery department due to lower limb fractures; following a thorough examination, he was diagnosed with a left tibia and fibular open fracture (Figure 1). He had significant bone loss in his tibia. Wound debridement and application of an external fixator were performed on admission. After 48 hours he underwent re-look surgery.

**Figure 2:**
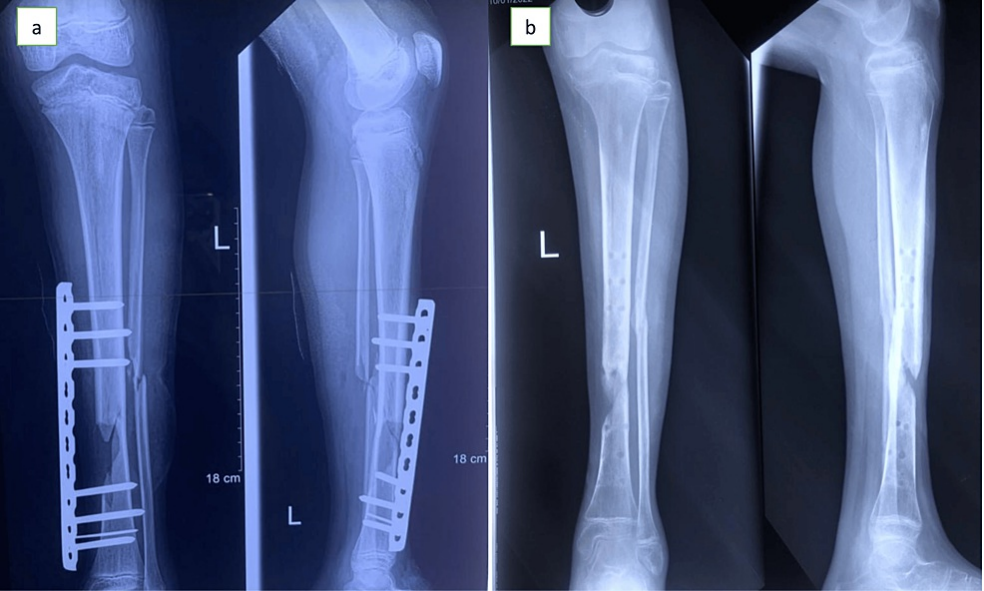
Radiological images following surgery showing bone alignment using an external fixator (a) Immediate X-ray achieving the desired alignment of the bone; (b) Post external fixator removal X-ray showing callus formation on both tibia and fibula

At the initial surgery, the locking compression plate as an external fixation method was used. The initial steps are similar to using an exchange fixator instead of a traditional external fixator. After positioning the patient on a radiolucent operating table, all the bite lacerations were extended, and debridement and pulsed lavage were performed while the patient was under general anesthesia. The chosen locking plate was placed at the fracture site. Real-time imaging is then employed to ensure proper plate positioning. Following holes were bored over locking drill guides through stab incisions where the overlaying soft tissue envelope remained intact. And screws were then implanted through them. Six screws were inserted with care to avoid using them at the fracture site. The entire structure was then studied once more with further X-ray imaging (Figure 2). Once the alignment was deemed satisfactory, the remaining soft tissue defect and screw sites were treated in the conventional way.

**Figure 3:**
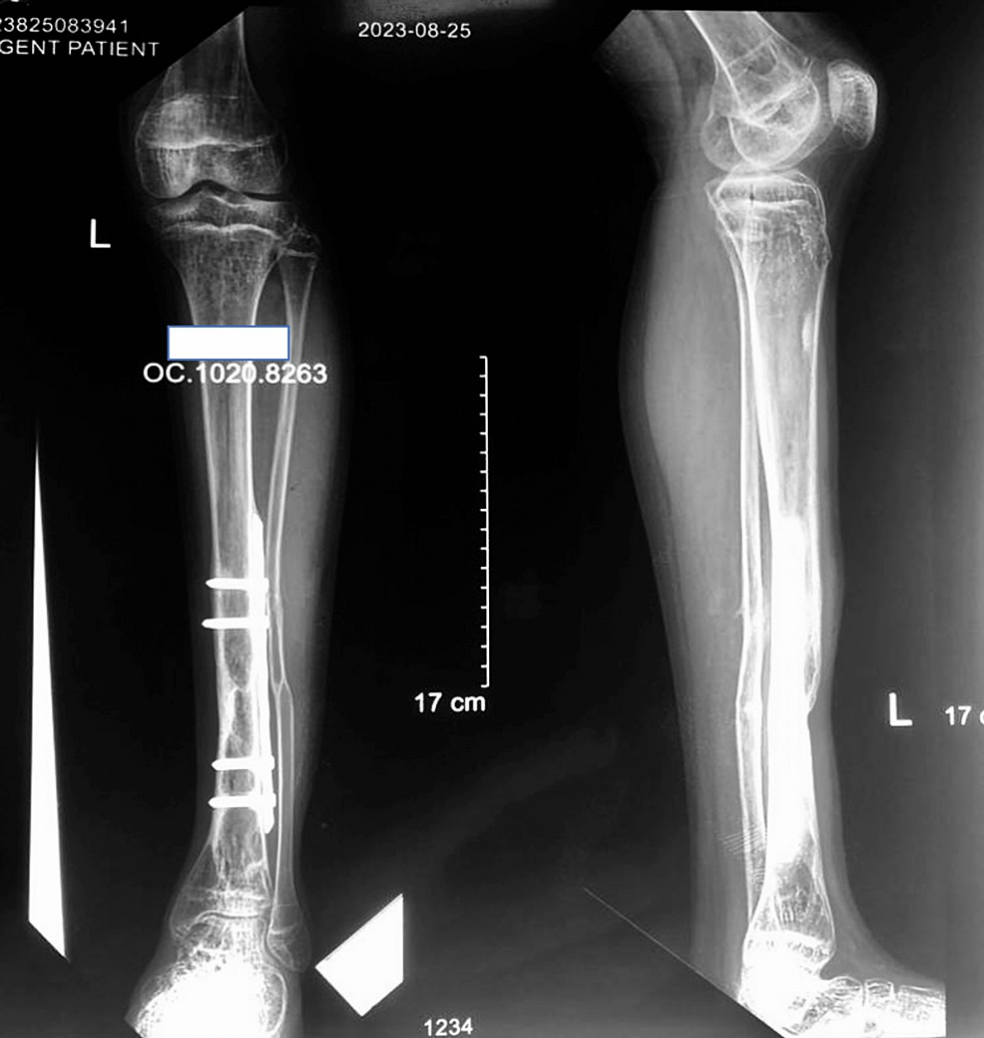
A completely united fracture in a 1-year follow-up

After 6 weeks, once all the wounds healed, the external fixator was removed. The bony union had been noted by the time he had progressed to partially weight-bearing with a walking aid. However, because of the bone loss at the tibia, the risk of re-fracture was anticipated. After 3 weeks of removal of the externally applied locking compression plate, he was taken into the theatre and applied for a locking plate with minimal invasive technique (Figure 3). Then he was allowed to full weight bearing. He was discharged from the follow-up after being fully mobilized with non-performance restrictions.

## Discussion

Surgeons face substantial difficulty in treating severe crocodile bites for a variety of reasons. At first glance, crocodile bite injuries are often caused by a mix of crushing and degloving, resulting in many wounds caused by the creature's long rows of teeth. Even modest puncture wounds can conceal underlying muscle injury, which may be accompanied by fractures. The injury's complexity needs careful assessment and treatment. Second, the wounds are contaminated with crocodile oral flora, which includes an uncommon combination of bacteria. These germs can cause significant local tissue damage and hemolysis, worsening the healing process even further.

Third, the accessibility to a center with orthopedic facilities. These delays can considerably impact the overall rate of complications connected with these injuries. However, the facilities available in the low-income setting are also challenging for surgeons from developing countries [5].

Resuscitation, tetanus prophylaxis, empirical wide-spectrum antibiotics, and wound debridement under general anesthetic are all part of the initial therapy [6]. If there is an accompanying fracture, the use of an external fixator considerably aids in continuous wound care. In our case, a locking plate is used as an external fixator after autoclaving. 

Due to a lack of resources, serious injuries, and logistical issues, surgeons in low-income countries (LIC) are forced to improvise during surgery and employ implants "off-label." The behavior of locking screws and plates differs from general internal fixators. They more closely resemble "internal" external skeletal fixators [7]. Although the threads of the screw shaft engage the bone, the interface between bone and screw is unrelated to the plate and screw attachment. The interface between the screw head and the plate in locking plates is what holds the plate to the screw. The bone retains its location relative to the plate while the screw is tightened; it is not pushed up to it. This means that exact anatomical contouring is not necessary [8]. Locking plate constructions with enhanced angular rigidity place more strain on the screw. This behavior of the internal-external skeletal fixation of the locking plate was used in this case due to limited resources. Improvisation of a used locking plate after autoclaving has allowed the bone to heal and align in its supposed way [9,10]. 

In addition to the inherent difficulties posed by injuries resulting from crocodile bites, surgeons encounter further challenges in managing these cases due to limited resources. This is particularly evident in regions with low-income settings where comprehensive medical facilities may be scarce [11]. The scarcity of resources can encompass a range of factors, including access to specialized equipment, availability of surgical supplies, and even the availability of trained medical personnel. Moreover, access to advanced imaging technologies like CT scans or MRI machines may be limited, potentially impeding accurate diagnosis and treatment planning. In some cases, surgeons may have to rely on less advanced imaging techniques or clinical assessment alone [12]. 

Overall, while surgeons strive to provide the best possible care for patients with crocodile bite injuries, the challenges arising from resource limitations highlight the need for targeted interventions and support to ensure optimal outcomes for affected individuals, especially in underserved areas.

## Conclusions

Surgeons grappling with severe crocodile bite injuries face a multifaceted challenge. These injuries, characterized by a combination of crushing and degloving, result in complex wounds, often concealing underlying muscle damage and potential fractures. In tandem with the intrinsic difficulties of treating crocodile bite injuries, the scarcity of resources further compounds the challenges faced by surgeons. This is acutely evident in regions marked by low-income settings, where the availability of comprehensive medical facilities is often inadequate. The dearth of resources encompasses factors ranging from access to specialized equipment to the availability of surgical supplies and trained medical personnel. However, it is imperative to note that even with an improvised technique bone alignment was achieved without any need for re-do surgery, making using a locking compression plate as an external fixator equivalent to standard practice. 

In conclusion, while surgeons are dedicated to delivering the highest standard of care for patients afflicted by crocodile bite injuries, the constraints imposed by resource limitations underscore the imperative for targeted interventions and support. This is particularly crucial in underserved areas, where tailored approaches are indispensable to achieving optimal outcomes for affected individuals.
